# Effect of Xeroderma pigmentosum complementation group F polymorphisms and H.pylori infection on the risk of gastric cancer

**DOI:** 10.12669/pjms.293.3480

**Published:** 2013

**Authors:** Li-li Zhang, Fang Yang, Ting-min Chang, Shu-ping Dong, Ying-ying Fan, Ting Lu

**Affiliations:** 1 Li-li Zhang, Department of Gastroenterology, The First Affiliated Hospital of Xinxiang Medical University, Weihui, China.; 2 Fang Yang, Department of Gastroenterology, The First Affiliated Hospital of Xinxiang Medical University, Weihui, China.; 3 Ting-min Chang, Department of Gastroenterology, The First Affiliated Hospital of Xinxiang Medical University, Weihui, China.; 4 Shu-ping Dong, Department of Gastroenterology, The First Affiliated Hospital of Xinxiang Medical University, Weihui, China.; 5 Ying-ying Fan, Department of Gastroenterology, The First Affiliated Hospital of Xinxiang Medical University, Weihui, China.; 6 Ting Lu, Department of Gastroenterology, The First Affiliated Hospital of Xinxiang Medical University, Weihui, China.

**Keywords:** Xeroderma pigmentosum complementation group F, Single Nucleotide Polymorphism, Gastric cancer, H.pylori

## Abstract

***Objective:*** We conducted a case-control study by genotyping three potential functional SNPs to assess the association of Xeroderma pigmentosum complementation group F (XPF) polymorphisms with gastric cancer susceptibility, and role of XPF polymorphisms in combination with *H.pylori *infection in the risk of gastric cancer.

***Methodology:*** A hospital case-control study was conducted. A total of 331 patients with gastric cancer and 355 controls were collected. Three SNPs of XPF, XPF rs180067, rs1799801 and rs2276466, were genotyped by Taqman real-time PCR method with a 7900 HT sequence detector system.

***Results:*** The gastric cancer patients were more likely to have smoking habit, a family history of cancer and H.pylori infection. We did not find the significant difference in the genotype distributions of XPF rs180067, rs1799801 and rs2276466 between cases and controls. Multivariate logistic analysis showed a non-significant decreased risk in patients carrying rs180067 G allele, rs1799801 T allele or rs2276466 T allele genotypes. The stratification by H.pylori infection was not significantly different in polymorphisms of XPF rs180067, rs1799801 and rs2276466.

***Conclusion:*** There was no evidence that polymorphisms in rs180067, rs1799801 and rs2276466 significantly affect the risk of gastric cancer. Further large sample size studies are strongly needed to validate their association.

## INTRODUCTION

Worldwide, gastric cancer is the second leading cause of death from cancer, with an estimated one million new cases in 2008 (988 000 cases), accounting for 8% of all cancer-related death worldwide. More than 70% of all gastric cancer cases occurred in developing countries, and approximately half of all cases occur in China.^1^ Many epidemiological studies suggest that Helicobacter pylori (H. pylori) infection is one of the most important risk factors for gastric cancer. It is estimated almost 50% of the world’s population are infected with H.pylori, but only about 1% of them occur gastric cancer.^2^^,^^3^ Therefore, the genetic and environmental factors may influence the susceptibility to gastric cancer.^4^^,^^5^

DNA repair plays a key role in keeping the function of the stability and integrity of human genome. It is well known that there are five main DNA repair pathways with more than 130 genes, of which the nucleotide excision repair (NER) pathway plays a crucial DNA repair mechanism by removing various DNA lesions caused by UV radiation and some chemical agents, such as bulky adducts, cross links, oxidative DNA and alkylating damage and thymidine dimers.^6^^-^^8^ It is reported that xeroderma pigmentosum (XP) complementation groups in human, XPA to XPG, represent the rate-limiting proteins in the NER pathway.^9^

The XPF gene is located on chromosome 16p13.12, contains 11 exons and spans approximately 28.2 kb, and plays a key role in the 5’ incision made in the NER pathway, and function as removing DNA interstrand cross-links and DNA double-strand breaks.^10^^,^^11^ It is reported that polymorphisms in several SNPs of XPF alter genetic susceptibility to cancer, such as colorectal cancer, breast cancer, lung cancer and prostate cancer as well.^12^^-^^15^ However, there is still no evidence on the association between XPF polymorphisms and gastric cancer risk in Chinese population.^16^ Therefore, we conducted a case-control study by genotyping three potential functional SNPs to assess the association of XPF polymorphisms with gastric cancer susceptibility, and role of XPF polymorphisms in combination with *H. pylori *infection in the risk of gastric cancer.

## METHODOLOGY

***Subjects:*** All the subjects were recruited from the First Affiliated Hospital of Xinxiang Medical University between January 2008 and December 2010. A total of 402 patients with newly histopathologically confirmed primary gastric cancer, including gastric cardia adenocarcinoma and non-gastric cardia adenocarcinoma, were included in our study. Of 402 patients, 331 patients were willing to participate into our study, with a participation rate of 82.3%. We excluded those who suffered from secondary or recurrent tumors. A total of 377 controls were selected from the same hospital during the same time period, and 355 controls wanted to participate into our study. Controls were outpatients from Surgical Department, Plastic Surgery Department and ENT Department as well. Controls who had a history of cancer and digestive tract disorders were excluded. All patients were asked to provide 5ml blood samples for DNA extraction. This study was approved by the ethics committees of the First Affiliated Hospital of Xinxiang Medical University, and informed consent was obtained from all recruited cases and controls.

***H.pylori infection Diagnosis*** *:* The H. pylori infection status was determined by the method of enzyme linked immunoabsorbent assay (ELISA) from 5 ml blood. H.pylori IgG antibodies (HpIgG Ab) were measured using commercially available kit (Genesis Diagnostics, Cambridgeshire, UK) according to the manufacturer’s instructions.

***Genotyping of XPF:*** We selected potential functional SNPs of interested XPF from Database of single nucleotide polymorphisms (SNPs) of NCBI (http://www.ncbi.nlm.nih.gov/) and SNPinfo (http://snpinfo.niehs.nih.gov/) with the following criteria: (1) the minor allele frequency ≥10% of the Chinese population; (2) influencing the microRNA binding sites activity. Genomic DNA was extracted using the buffy coat fraction of each blood sample with a Qiagen Blood Kit (Qiagen, Chastworth, CA) according to the manufacturer’s instructions. DNA purity was conducted by spectrophotometric measurement of absorbance at 260 and 280 UV spectrophotometers. Finally, the three SNPs of XPF were chose in our study, including rs180067, rs1799801 and rs2276466. Genotyping of the three SNPs was performed by the method of Taqman real-time PCR with a 7900 HT sequence detector system (Applied Biosystems, Foster City, CA). For quality control, we randomly selected 10% of the samples to repeat genotyping, and we gained a reproducibility of 100%.

***Statistical analysis:*** Statistical analysis was performed by SPSS version 16.0 statistical software (SPSS, Chicago, IL, USA). Continuous variables were expressed as mean ± standard deviation (SD), while categorical variables were shown as percentages. The demographic and clinical characteristics were compared between cases and controls by χ^2^ test or student’s test. Differences in frequency distributions of the selected SNPs between cases and controls were evaluated by χ^2^ test. Hardy-Weinberg equilibrium in controls was acalculated by χ^2^ test. Adjusted odds ratios (OR) and their 95% confidence intervals (95% CI) was used to assess the association between the polymorphisms in selected SNPs and gastric cancer risk by multivariate logistic regression model. The interaction of the three SNPs with H.pylori was also estimated by spearman correlation analysis. Statistical significance was set at *P<0.05 *and all tests were two-sides.

## RESULTS

The demographic and clinic characteristics of the selected cases and controls are shown in Table-I. The mean ages of the 331 cases with gastric cancer and 355 controls were 55.7±8.3 and 56.3±8.5 years, respectively. We did not find significant differences in the ages or sex distributions between cases and controls (*P* > 0.05). There was no significant difference in the drinking status between the two groups. However, the gastric cancer patients were more likely to have smoking habit, a family history of cancer and H.pylori infection (*P* < 0.05). Of the cancer cases, 54.6% were intestinal, and 40.8% were diffuse. 54.3% of the cases were early gastric cancer.

The genotype distributions of XPF rs180067, rs1799801 and rs2276466 in cases and controls is summarized in Table-II. All the genotype frequencies of selected SNPs in controls were in line with the Hardy-Weinberg equilibrium. However, we did not find the significant difference in the genotype distributions of XPF rs180067, rs1799801 and rs2276466 between cases and controls. Multivariate logistic analysis was conducted to evaluate the effect of XPF rs180067, rs1799801 and rs2276466 on the risk of gastric cancer. Using the wide-type genotype as the reference genotype, we found a non-significant decreased risk in patients carrying rs180067 G allele, rs1799801 T allele or rs2276466 T allele genotypes.

A further association analysis was conducted to evaluate the association of XPF rs180067, rs1799801 and rs2276466 with risk of gastric cancer by stratifying H.pylori infection. However, the stratification by H.pylori infection was not significantly different in polymorphisms of XPF rs180067, rs1799801 and rs2276466. The three XPF SNPs did not show significant interaction with H.pylori infection (*P *for interaction was 0.35).

## DISCUSSION

To the best of our knowledge, this study is the first attempt to evaluate the potential association between the three selected SNPs of XPF and risk of gastric cancer in Asian population. We did not find the significant association between the three selected SNPs and risk of gastric cancer. Moreover, we did not find the H.pylori infection modified the association between the selected SNPs and gastric cancer risk. Although the published genome-wide associated study has not showed XPF variants was an susceptibility loci, it is difficult to evaluate the effects of the genotypes for the potentially functional SNPs in XPF in the genome-wide associated study due to not enough samples. In the present study, we found the polymorphisms in rs180067, rs1799801 and rs2276466 was not associated with gastric cancer risk, and no interaction was found between H.pylori.

**Table-I:** Distributions of demographic and clinical characteristics

*Characteristic*	*Cases*	*%*	*Controls*	*%*	*P*
	*N=331(%)*		*N=355(%)*		
Age, yr (Mean±SD)	55.7±8.3		56.3±8.5		0.82
Male	214	64.6	210	59.1	0.14
Family history of cancer	18	5.3	2	0.6	<0.001
Smoking status					
Ever	108	32.6	86	24.2	0.015
Never	223	67.4	269	75.8	
Drinking status					
Ever	123	37.2	113	31.7	0.215
Never	208	62.8	242	68.3	
H.pylori infection(positive)	195	58.9	182	51.4	0.04
Histological type of gastric cancer					
Intestinal	181	54.6			
Diffuse	135	40.8			
Mixed	15	4.6			
Stage of gastric cancer	0				
Early gastric cancer	180	54.3			
Advanced gastric cancer	151	45.7			

**Table-II:** Frequency distribution of XPF SNPs polymorphisms and their association with gastric cancer risk

*Genotype*	*Cases* *N=337*	*%*	*Controls* *N=347*	*%*	*OR (95% CI)* ^1^	*P*
rs180067						
CC	257	77.5	261	73.5	1.0(Reference)	-
CG	57	17.1	69	19.4	0.94(0.56-1.26)	0.37
GG	18	5.4	25	7.10	0.73(0.37-1.43)	0.33
rs1799801						
CC	223	67.3	232	65.4	1.0(Reference)	-
CT	68	20.6	80	22.5	0.88(0.60-1.31)	0.52
TT	40	12.1	43	12.1	0.95(0.59-1.59)	0.89
rs2276466						
CC	220	66.4	223	62.7	1.0(Reference)	-
CT	87	26.4	101	28.5	0.87(0.61-1.25)	0.44
TT	24	7.2	31	8.8	0.78(0.43-1.43)	0.40

Adjusted for sex, age, drinking, smoking, H.pylori infection and family history of cancer

**Table-III:** Subgroup analysis by H.pylori infection status

*Genetic polymorphisms*	*H.p* *ylori infection [OR(95% CI)]* ^1^	
	*Positive*	*Negative*
rs180067		
CC	1.0(Reference)	1.0(Reference)
CG	1.13(0.41-1.40)	0.86(0.52-1.31)
GG	0.89(0.35-1.58)	0.66(0.30-1.53)
rs1799801		
CC	1.0(Reference)	1.0(Reference)
CT	0.93(0.68-1.51)	0.83 (0.50-1.44)
TT	1.02(0.54-1.63)	0.92(0.54-1.53)
rs2276466		
CC	1.0(Reference)	1.0(Reference)
CT	1.07(0.77-1.48)	0.80(0.57-1.28)
TT	0.95(0.59-1.34)	0.72(0.41-1.47)

1. Adjusted for sex, age, drinking, smoking and history of cancer

The XPF rs2276466 and rs3136038 was found to be associated with a reduced risk of squamous cell carcinoma of the head and neck in a previous study.^17^ This study indicated the GF genotype of rs2276466 was significantly associated with a decreased risk of head and neck cancer, and TT genotype of rs3136038 showed a borderline significant decreased cancer risk.^17^ A previous meta-analysis indicated that XPF-rs1799801 may be associated with a reduced cancer risk in Caucasian population, and XPF-rs1800067 was related to a decreased risk of pancreatic cancer. However, a few studies have investigated the association between XPF SNPs and risk of gastric cancers, and only two studies reported their association.^18^^,^^19^ One study conducted in China analyzed the two XPF functional SNPs, rs2276466C>G and rs6498486A>C, with risk of gastric cancer, and reported that no functional XPF SNPs contribute to risk of gastric cancer.^18^ Another study also conducted in China showed no significant association of XPF-357A/C with the risk of gastric cancer in a population of a high-incidence region of China.^19^ Our study also found no significant association between polymorphisms in rs180067, rs1799801 and rs2276466 and gastric cancer risk, which are similar to the previous results. However, our findings need to be confirmed by large-scale studies.

There were some limitations in our study. First, the patients in our study were selected from one hospital, which may be lack of presentation, and have selection and information bias. Second, we only analyzed three potential SNPs of XPF, which did not cover all the SNPs of XPF and may have selection bias. We did not have enough information on other environmental factors due to a retrospective study design, such as lifestyle and dietary habits. Finally, the relatively small sample size may have not enough power to find the association between polymorphisms in SNPs and gastric cancer risk.

## CONCLUSION

In conclusion, our study did not find evidence that the polymorphisms in rs180067, rs1799801 and rs2276466 significantly affect the risk of gastric cancer. Further large sample size studies are strongly needed to validate their association.
